# Pregnancy Outcomes in HIV-Infected Women Receiving Long-Term Isoniazid Prophylaxis for Tuberculosis and Antiretroviral Therapy

**DOI:** 10.1155/2013/195637

**Published:** 2013-03-07

**Authors:** Allan W. Taylor, Barudi Mosimaneotsile, Unami Mathebula, Anikie Mathoma, Ritah Moathlodi, Irene Theebetsile, Taraz Samandari

**Affiliations:** ^1^Division of HIV/AIDS Prevention, Centers for Disease Control and Prevention (CDC), National Center for HIV/AIDS, Viral Hepatitis, STD, and TB Prevention, 1600 Clifton Road, NE MS E-45, Atlanta, GA 30333, USA; ^2^BOTUSA, CDA Gaborone and Francistown, Plot 14818 Lebatlane Road, Phase One, Gaborone, Botswana; ^3^Division of Tuberculosis Elimination, CDC, 1600 Clifton Road NE, Mailstop E-45, Atlanta, GA 30333, USA

## Abstract

*Objective*. While 6- to 12-month courses of isoniazid for tuberculosis prevention are considered safe in pregnant women, the effects of longer-term isoniazid prophylaxis or isoniazid in combination with antiretroviral therapy (ART) are not established in human-immunodeficiency-virus-(HIV-) infected women who experience pregnancy during the course of therapy. *Design.* Nested study of pregnancy outcomes among HIV-infected women participating in a placebo-controlled, TB-prevention trial using 36 months daily isoniazid. Pregnancy outcomes were collected by interview and record review. *Results*. Among 196 pregnant women, 103 (52.6%) were exposed to isoniazid during pregnancy; all were exposed to antiretroviral drugs. Prior to pregnancy they had received a median of 341 days (range 1–1095) of isoniazid. We observed no isoniazid-associated hepatitis or other severe isoniazid-associated adverse events in the 103 women. Pregnancy outcomes were 132 term live births, 42 premature births, 11 stillbirths, 8 low birth weight, 6 spontaneous abortions, 4 neonatal deaths, and 1 congenital abnormality. In a multivariable model, neither isoniazid nor ART exposure during pregnancy was significantly associated with adverse pregnancy outcome (adjusted odds ratios 0.6, 95% CI: 0.3–1.1 and 1.8, 95% CI 0.9–3.6, resp.). *Conclusions*. Long-term isoniazid prophylaxis was not associated with adverse pregnancy outcomes, such as preterm delivery, even in the context of ART exposure.

## 1. Introduction 

Tuberculosis (TB) is a major cause of morbidity and mortality for HIV-infected persons [1]. A clinical trial was recently completed in Botswana, in which a 36-month course of isoniazid prophylaxis against TB (IPT) was highly efficacious in reducing the risk of TB in tuberculin-skin-test-positive adults [2] when compared with a shorter-term regimen. Based upon this and other evidence [3], the World Health Organization recommended that in countries with high TB transmission, national health programs consider providing 36-month isoniazid prophylaxis for persons living with HIV [4]. The HIV epidemic disproportionately affects women in developing countries [5]. As availability of antiretroviral medications for therapy (ART) and for prevention of mother-to-child HIV transmission (PMTCT) has been rapidly scaled up [6], and with increasing health, pregnancy is common. While 6- to 12-month courses of isoniazid are considered safe in pregnancy [7], our objective was to describe the effects of longer-term isoniazid prophylaxis or simultaneous isoniazid and ART in pregnant HIV-infected women.

## 2. Methods 

### 2.1. Study Population

A cohort of 1995 HIV-infected adults was enrolled at eight clinics located in Francistown and Gaborone, Botswana, from 2004 to 2006. Details of enrolment are provided elsewhere [8]. This double-blind, randomized, placebo-controlled trial gave participants 6 months of isoniazid (300–400 mg/day) plus vitamin B6 (25 mg/day), after which either isoniazid or placebo (1 : 1) was provided for 30 months longer. Participants returned monthly for medication refills. Per national policy at the time, if a woman became pregnant and was not already receiving ART, she was referred to an antenatal clinic for free short-course zidovudine (AZT) prophylaxis from week 34 of gestation (if her CD4+ T-cell count was >200/mm^3^) or ART if her CD4+ T-cell count was <200/mm^3^. Pregnancy outcome data were collected by maternal interview and from antenatal clinic records.

### 2.2. Definitions

Severe hepatitis was defined as elevation of liver enzymes to >5 times the upper limit of normal. Severe rash was defined as generalized erythroderma, eruption, or desquamation covering ≥50% of body surface area. Adverse events were determined by a blinded, independent committee to be possibly, probably, definitely, or not associated with the study medication. We defined adverse pregnancy outcome (AO) as any of the following: preterm delivery (estimated gestational age (EGA) ≤37 weeks at birth), low birth weight (<2500 g), stillbirth (delivery of an infant with no signs of life at ≥28 weeks EGA), spontaneous abortion (spontaneous termination of pregnancy <24 weeks EGA), neonatal mortality (death of term infant within 28 days of delivery), or any noted congenital abnormality. We defined ART as therapy with ≥3 antiretroviral drugs from ≥2 classes provided for treatment of HIV disease. The pregnancy outcomes analysis included only the first-born infant of the first pregnancy experienced by women during the trial pregnancies completed by February 19, 2009, and only included women who registered for PMTCT.

### 2.3. Statistical Methods

We performed univariate, bivariate, and multivariable analysis to investigate associations between AO, isoniazid exposure, and ART exposure in pregnancy. Continuous variables were compared using *t*-tests or nonparametric tests. We computed bivariate odds ratios to test crude associations of *a priori* interest. Multivariable logistic regression models were employed to explore associations of interest while controlling for potential confounders, including maternal CD4+ T-cell count nearest the last normal menstrual period (LNMP), maternal body mass index (BMI < 18.5), and age. Analyses were conducted using SAS v9.3 (SAS Institute Inc., Cary, NC, USA).

### 2.4. Ethical Considerations

All participants provided informed consent. The protocol was approved by Botswana and Centers for Disease Control and Prevention (CDC) ethics committees. The trial was registered at http://www.clinicaltrials.gov/, NCT00164281.

## 3. Results

Seventy-two percent of the 1995 enrollees were female; their median age was 32 years; their median CD4+ count was 297 cells/mm^3^, and 47% initiated ART during the 36-month period of observation. Of the 1436 women in the study, 721 (50.2%) were randomized to 6 months of IPT; 715 women (49.8%) were randomized to 36 months of IPT. A total of 268 pregnancies were observed during the trial. We excluded 29 women who were still pregnant at the time the dataset was closed or who exited the trial prior to the end of pregnancy, 23 repeat pregnancies, and 20 women without PMCT regimen data, leaving a total of 196 pregnancies in the analysis dataset (98 per arm). Some participants received placebo after 6 months of IPT or stopped taking isoniazid while remaining under observation in the trial, resulting in some women not receiving isoniazid while pregnant. Of women in the 6-month isoniazid arm, 20 were exposed to isoniazid during pregnancy, and 39 were exposed to ART. Of women in the 36-month isoniazid arm, 83 were exposed to isoniazid in pregnancy, and 34 were exposed to ART.

Overall, 103/196 (52.6%) women were exposed to isoniazid during some part of pregnancy, with 102 beginning in the first trimester, and 68% of these women had continuing exposure throughout pregnancy (Table 1). None of the women receiving isoniazid had severe hepatitis or rash either during pregnancy or in the postpartum period. Among these 103 women, 94 had begun isoniazid before pregnancy; the median duration of isoniazid receipt before the last menstrual period for these women was 341 days (range 1–1095 days).

All women were exposed to antiretroviral drugs during pregnancy: 73 (37%) received ART, and the remainder received either short-course zidovudine (121) or zidovudine/lamivudine (AZT/3TC) (2). Of 73 women receiving ART during pregnancy, the most (64/73, 88%) received AZT/3TC and nevirapine; 3 received lopinavir/ritonavir-based regimens; 6 received other combinations. Women taking ART had significantly lower CD4+ T-cell counts than those taking short-course prophylaxis (median CD4+ count 239 versus 452, Wilcoxon rank-sum *P* < 0.0001). Of the 73 women receiving ART, 62% did so during all 3 trimesters. For the 47 women who began taking ART before pregnancy, the median duration of ART before pregnancy was 347 days (range 21–2221 days). Twenty-six initiated ART during pregnancy.

Two pregnant women developed TB symptoms during their pregnancies. One started anti-TB treatment 3 months after having a live birth, and the other initiated anti-TB treatment a month after her LNMP and sustained a stillbirth 7 months later. Both successfully completed standard 6-month anti-TB treatment.

Final outcomes of the 196 pregnancies were 124 (63%) term live births; 42 (21.4%) premature deliveries, 8 (4%) low-birth-weight term infants, 11 (6%) stillbirths, 6 spontaneous abortions (3%), 4 neonatal deaths (2%), and 1 congenital abnormality (talipes equinovarus, Table 1).

In bivariate analysis (Table 2), ART receipt during pregnancy was associated with AO (unadjusted odds ratio (uOR) 2.0, 95% confidence interval (CI) 1.1–3.5), as was increasing maternal age (uOR 1.1 per year, 95%CI 1.0–1.1). Isoniazid exposure during pregnancy was not associated with increased odds of AO. Length of exposure to INH prior to pregnancy was also not associated with increased odds of AO when analyzed either as a continuous or categorical variable (data not shown).

In a multivariable model including all variables in Table 2, none was significantly associated with increased odds of AO except maternal age. Addition of a dichotomous variable representing maternal CD4 count <200 near delivery did not significantly change any model parameter, and this variable was not retained. A separate but otherwise identical model showed no association between any variable (including ART exposure) and the outcome of preterm delivery (not shown). Similarly, another separate analysis with the same covariates but with the antiretroviral drug exposure variable dichotomized as exposure prior to pregnancy or in the first trimester versus all others demonstrated no significant association with preterm delivery (not shown). 

## 4. Discussion

We observed no isoniazid-associated hepatitis or other severe isoniazid-associated adverse events in 103 women receiving isoniazid during pregnancy and/or immediately postpartum. Generally, isoniazid hepatitis occurs in the first months of treatment, as it did in the Botswana clinical trial [2]; pregnant women in this study had received isoniazid for a median of 341 days before pregnancy. In the earlier literature there was inconclusive evidence of potentially increased risk of isoniazid-associated hepatitis and death in pregnant or post-partum women [9, 10]. As they have a high risk of developing active TB, the CDC and American Thoracic Society recommend that IPT be initiated in HIV-infected women even during the first trimester (with routine monitoring of hepatic enzymes) [7]. Botswana policy is that, while women known to be pregnant should not initiate IPT, if they do become pregnant they should continue their course of therapy without monitoring hepatic enzymes. The overall risk of isoniazid-associated death observed in this trial—occurring in two nonpregnant women among 1995 persons who initiated isoniazid prophylaxis [2]—was no higher than the established estimated rate of hepatic death and hepatitis requiring liver transplantation of approximately 1/1000 in the United States [11].

Extensive use of isoniazid during pregnancy has indicated that although it readily crosses the placental barrier, the drug is not teratogenic even when given during the first trimester [12, 13]. We report one congenital abnormality (talipes equinovarus) in a child born to a woman who was taking isoniazid near conception. The background rate for this abnormality is unknown in Botswana; in the United States this rate has been estimated at approximately 1/1000 live births [14–16]. To date a prospective registry has found no apparent increase in the frequency of this condition among infants exposed prenatally to antiretroviral medications [17].

In this study we did not observe increased odds of adverse pregnancy outcomes among women receiving ART in pregnancy compared to women receiving short-course antiretroviral regimens. Previous studies offered mixed evidence of such an association. Studies from Europe found an association between ART (especially with protease inhibitor-based ART) exposure in pregnancy and preterm delivery [18, 19]. Similarly, a study in Botswana found increased odds of small-for-gestational-age infants among women taking ART [20]. A separate study of 71 antiretroviral drug-exposed pregnancies in Botswana found no difference in rates of early pregnancy loss or stillbirths between efavirenz- and nonefavirenz-exposed pregnancies; only one congenital abnormality was observed (unrelated to efavirenz exposure) [21]. Studies from the United States have not generally observed such an association [22, 23], except possibly for women starting ART before pregnancy or during the first trimester, compared to later initiation [24]. Given the known association between advanced HIV disease and preterm delivery [25, 26], it is possible that the borderline association between ART use and adverse birth outcome observed in the unadjusted analysis of the present study was due to confounding by maternal health status. This interpretation is supported by the disappearance of the association after controlling for maternal CD4+ count. 

This study provided a unique opportunity to examine pregnancy in the context of long-term INH exposure. Chief among its limitations was the small sample size, which limited the power of the study. Also, as antiretroviral medication use was not randomized, we were unable to rule out the existence of unmeasured confounders in our analysis of antiretroviral use and AO.

In summary, long-term prophylaxis with IPT appeared to be safe in this small secondary analysis, even when pregnancy was experienced by HIV-infected women during therapy and was not associated with adverse pregnancy outcomes.

## Figures and Tables

**Table 1:** Selected characteristics of subjects experiencing pregnancy during the Isoniazid Preventive Therapy Trial, Botswana, 2005–2008 (*n* = 196).

	No.	% or range
Characteristic (*n* = 196)		
Age (median, range)	28	19–39
CD4+ lymphocyte count nearest LNMP (median, range) in cells/mm^3^	368	41–1231
CD4+ lymphocyte count near LNMP <200 cells/mm^3^	31	16%
Maternal BMI (median, range)	22.9	15.6–36.2
Maternal BMI <18.5 near LNMP	17	19%
Pregnancy outcome date range		Nov 26 2005 to Feb 19 2009
Antiretroviral regimen in Pregnancy		
Antiretroviral therapy (≥3 drugs from ≥2 classes)	73	37%
AZT or AZT/3TC only	123	63%

Antiretroviral therapy exposure (*n* = 196)		
Number of women on ART prior to pregnancy	47	
Time on ART before pregnancy (median, range) in days*	347	21–2221
Timing of ART exposure during pregnancy		
First, second, and third trimesters	45	
First and second trimesters only	7	
Second and third trimesters only	8	
Third trimester only	8	
Timing unknown	5	

Isoniazid exposure (*n* = 103)		
Time on isoniazid before pregnancy (median, range) in days	341	1–1095
Exposed to isoniazid in pregnancy		
First trimester only	11	11%
First and second, trimesters only	21	20%
First, second, and third trimesters	70	68%
Second trimester only	1	1%
Any trimester (total)	103	100%

Pregnancy outcome (*n* = 196)		
Live birth	124	63%
Stillbirth	11	6%
Spontaneous (“inevitable”) abortion	6	3%
Neonatal death	4	2%
Premature	42	21%
Term low birth weight	8	4%
Congenital abnormality	1	1%
Maternal death	0	0%

3TC: lamivudine; ART: antiretroviral therapy; AZT: zidovudine; BMI: body mass index; IPT: isoniazid preventive therapy; LNMP: last normal menstrual period.

*Among women with ART start date prior to LNMP (*n* = 47).

**Table 2:** Association between adverse pregnancy outcomes and isoniazid and/or antiretroviral therapy during pregnancy in HIV-infected women, Isoniazid Preventive Therapy Trial, Botswana, 2005–2008 (*n* = 196).

Characteristic	Adverse outcome no. (%)	No adverse outcome no. (%)	uOR	95% CI	aOR	95% CI
Isoniazid exposure in pregnancy						
Yes	32 (31.1)	71 (68.9)	0.6	0.3–1.1	0.6	0.3–1.1
No	40 (43.1)	53 (57.0)	1.0		1.0	
Antiretroviral therapy regimen in pregnancy						
Antiretroviral therapy (≥3 drugs from ≥2 classes)	34 (46.6)	39 (53.4)	2.0	1.1–3.5	1.8	0.9–3.6
Zidovudine or zidovudine/lamivudine only	38 (31.0)	85 (69.1)	1.0		1.0	
Maternal CD4+ lymphocyte count nearest LNMP						
<200 cells/mm^3^	13 (41.9)	18 (58.1)	1.3	0.6–2.8	1.0	0.4–2.5
≥200 cells/mm^3^	59 (35.8)	106 (64.2)	1.0		1.0	
Maternal BMI near LNMP						
Underweight <18.5	9 (52.9)	8 (47.1)	2.0	0.7–5.5	2.4	0.8–7.1
Not underweight ≥18.5	63 (35.8)	113 (64.2)	1.0		1.0	
Maternal age (years)	—	—	1.1	1.0–1.1	1.1	1.0–1.2

BMI: body mass index; aOR: adjusted odds ratio (adjusted for all variables shown); CI: confidence interval; uOR: unadjusted odds ratio; LNMP: last normal menstrual period.
